# Hurricane María’s Precipitation Signature in Puerto Rico: A Conceivable Presage of Rains to Come

**DOI:** 10.1038/s41598-019-52198-2

**Published:** 2019-10-30

**Authors:** Carlos E. Ramos-Scharrón, Eugenio Arima

**Affiliations:** Department of Geography & the Environment and Lozano Long Institute of Latin American Studies, The University of Texas at Austin, Austin, TX USA

**Keywords:** Climate sciences, Natural hazards

## Abstract

The effects of global climate change on the intensity of tropical cyclones are yet to be fully understood due to the variety of factors that affect storm intensity, the limited time spans of existing records, and the diversity of metrics by which intensity is characterized. The 2017 North Atlantic hurricane season induced record-breaking economic losses and caused hundreds of fatalities, and for many represents a presage of what the future holds under warmer tropical sea surface temperatures. This article focuses on one such major hurricane, María, and answers the question of how this event compares to the historical record of tropical storms that have assailed the island of Puerto Rico since 1898. Comparisons relied on interpolated weather station total rainfall and maximum 24-h rainfall intensities. María proved to have the greatest 24-h rain intensities among all storms recorded in Puerto Rico, yielding maximum 24-h recurrence intervals greater than 250 years for about 8% of the island.

## Introduction

Tropical cyclones (TCs) are amongst Earth’s most damaging natural hazards and their future effects are expected to proliferate due to demographic shifts, and increased intensity and frequency of the most extreme events^1–3^. TC intensity scales, whether of ordinal (e.g., Saffir-Simpson)^4^ or continuous^5^ character, are best suited for wind or ocean wave/surge damage, yet many TC impacts are rain-induced^6^. Adequately contrasting at-a-site rainfall differences among various TCs is difficult because the spatial distribution of rainfall is highly variable and most weather records tend to be too brief for proper long-term analyses. However, site-specific understanding is now crucial in improving our ability to predict the local expressions of global climate change on TC rainfall^7^. Previous approaches have relied on remotely sensed or modeled composite rainfall assessments of the entire lifetime of individual TCs^8,9^, or at-a-site analyses of remotely sensed^10^ or weather station data^11–13^.

Worldwide, the maximum contributions of TCs to annual rainfall occur near 18°N^14^. This is where the island of Puerto Rico (PR) is located and where TCs have provoked copious rainfall^15^ responsible for large numbers of slope failures^16,17^, as well as world records in instantaneous, area-normalized peak flows^18,19^. Models predict higher TC rain intensities under most projected climate change scenarios for the North Atlantic^20–22^ and specifically for PR^23^. Evidence shows that the anomalous 2017 North Atlantic hurricane season was enhanced by the warmest sea surface temperature on record^24^ and that this is partly attributable to global warming trends^25,26^. Hurricane María (HM) was a borderline Category 4–5 major hurricane as it entered PR on 20-Sep-2017^27^. HM’s low pressure center remained on land for eight hours, leaving a catastrophic legacy of human fatalities^28^, socio-economic loss^29,30^, geomorphic change^31,32^, and ecosystem impact^33,34^. This study answers the simple question of whether HM’s total and maximum 24-hr rainfall intensities (TR and INT, respectively) signify an anomaly relative to all 60 other significant TCs that have affected PR since 1899. Proof of HM as an irregular reference for TC rainfall in PR should serve as encouragement for promptly enforcing fundamental changes in TC preparedness for the Caribbean and elsewhere.

PR is the smallest and easternmost of the Greater Antilles. The island’s physiography consists of coastal lowlands, a northern karstic belt, and uplands consisting of three main units: Cordillera Central, Sierra de Cayey, and Sierra de Luquillo (Supplemental Fig. 1)^35^. The bulk of the annual rainfall occurring from May to December is influenced by low-pressure easterly waves, some of which organize into TCs^36^. Recurrence intervals for any TC to make landfall in PR is about 5 years^15^ but direct hits by major hurricanes (>3 Saffir-Sampson scale) have occurred about once every 50 to 60 years^37^.

## Results

### Total rainfall

**Figure 1:**
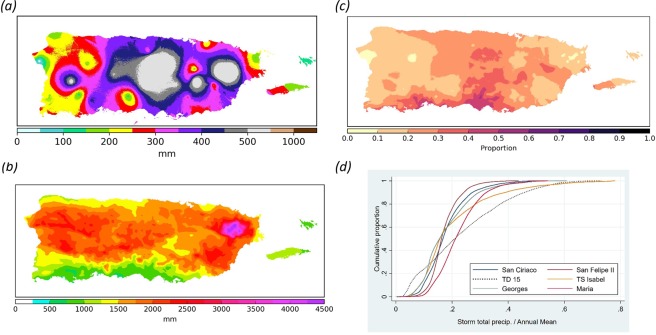
Hurricane María’s total rainfall versus annual normal rainfall. (**a**) Spatial distribution of total rainfall in mm during Hurricane María based on co-krigging analyses of rainfall recorded between the 19^th^ and the 21^st^ of September 2017 at 51 weather stations; (**b**) Mean annual rainfall for Puerto Rico [Prism Climate Group]; (**c**) Ratio of María total rainfall to mean annual rainfall to display the relative contribution of annual rainfall associated to María; (**d**) Cumulative distribution curves for the proportion of annual rainfall caused by the six tropical cyclones with the highest total rainfall values.

HM’s maximum at-a-station observed TR (733 mm) ranks 5^th^ relative to all other 60 TCs (Supplemental Table 1). The overall maximum weather station TR (976 mm) occurred during tropical depression #15 in 1970 (TD15) over a five-day period. Interpolations of the weather station data based on co-krigging analyses showed that HM equaled <1–46% of annual normal rainfall over the entire island (median = 21%; Fig. 1). The median ratio is within the 20–30% range previously described for TC-associated rainfall for PR^12^ and for the Northern Caribbean^11^. Therefore, HM had one of the highest TRs ever recorded in PR but its signature was not atypical when compared to previously recorded TCs.

**Figure 2:**
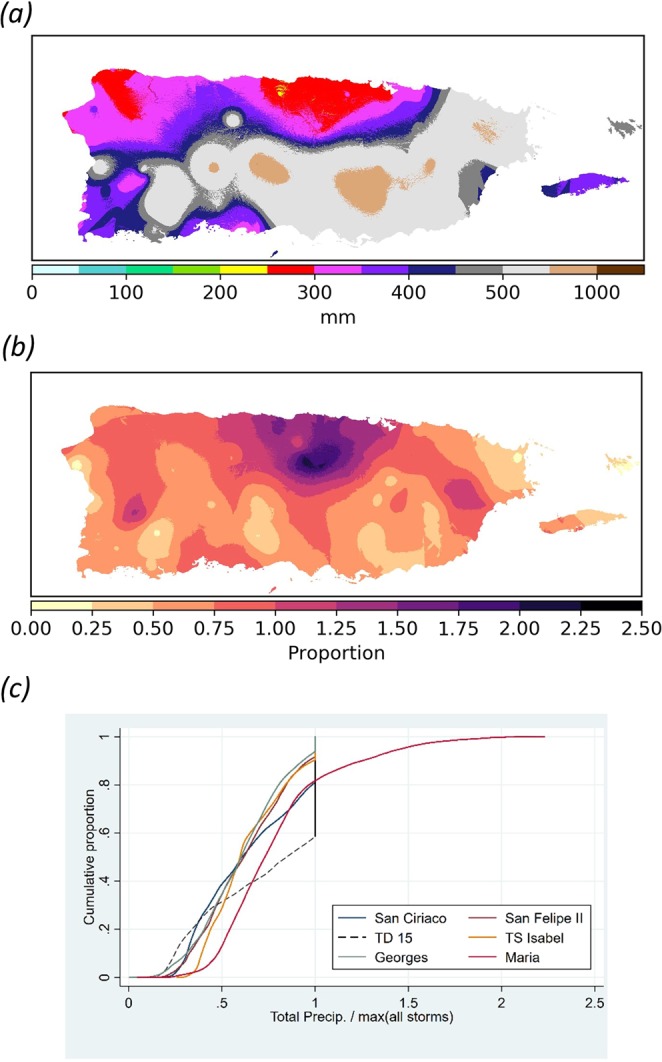
Hurricane María’s total rainfall versus previous maximums. (**a**) Overall maximum interpolated rain totals for all tropical cyclones from 1898 to 2017 except Hurricane María; (**b**) Ratio of María’s interpolated total rainfall to the maximum interpolated total rainfall for all other tropical cyclones (ratio of Figs 1a to 2a) [values ≥ 1 denote locations where María represents the historical maximum total rainfall]; (**c**) Cumulative distribution of the ratio of storm total rainfall to the maximum total rainfalls prior to María for the six tropical cyclones with the highest total rainfall values.

Hierarchical clustering analyses (Supplemental Method 1) of the interpolated station-specific observations indicates that HM and TD15 are essentially tied as having the highest PR-wide median TRs of all 61 TCs (~380 mm) (Fig. 2; Supplemental Fig. 2 and Table 2). During HM, 96% and 10% of the island received an excess of 200 mm and 500 mm of rain, respectively. Highest TRs during HM occurred in the western and central Cordillera Central, the western end of Sierra de Cayey, and in the Sierra de Luquillo. Similar to Hurricane Georges (1998), HM’s rainfall was the highest TC-related TR ever recorded for about 20% of PR’s landmass and this is half of that for TD15 (Fig. 2c).

### 24-hr rainfall intensity

**Figure 3:**
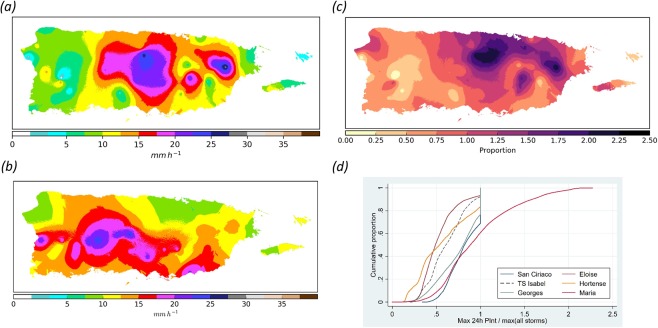
Hurricane María’s 24-h rainfall intensity signature. (**a**) Spatial distribution of 24-h rainfall intensities in mm h^−1^ during Hurricane María based on co-krigging analyses; (**b**) Overall maximum interpolated rain intensities of all tropical cyclones from 1898 to 2017 except María; (**c**) Ratio of María’s interpolated 24-h rainfall intensities to the maximum interpolated rain intensities for all other tropical cyclones (ratio of **a** to **b**) [values ≥ 1 denote where María represents the historical maximum 24-h rainfall intensity]; (**d**) Cumulative distribution of the ratio of 24-hr rainfall intensities to maximum rain intensities prior to María for the six storms with highest rain intensities.

Maximum at-a-station INT during HM was 28.3 mm hr^−1^ (682 mm in 24-h) in the northeastern portions of the island and this represents the overall maximum of the entire historical record (Supplemental Table 1). HM’s median, island-wide interpolated INT was 12.1 mm hr^−1^ (290 mm; Fig. 3). About 20% of the island during HM had INTs exceeding 16.7 mm hr^−1^ (400 mm) and these concentrated in the Sierra de Luquillo and in central PR along a south-to-north trending 40–50 km wide area.

Hierarchical clustering analyses demonstrated that the interpolated island wide INT for HM was distinct from all other storms. HM’s island-wide median INT was 13% higher than that of the second ranked TC (the 1899 Huracán San Ciriaco; median = 10.7 mm hr^−1^) (Supplemental Fig. 3 and Table 3). Prior to HM, Hurricanes San Ciriaco and Georges held record INTs for 31% and 23% of the island, respectively. During HM, 39% of PR’s land surface had INTs previously undocumented for any of the other 60 TCs (Fig. 3d). These areas concentrated in the central and eastern portions of the island with some small patches in the northwest coastal plains and in the southwestern portions of the Cordillera Central. About 13% of PR had INTs that exceeded previous records by at least 1.5 times.

### Recurrence interval

**Figure 4:**
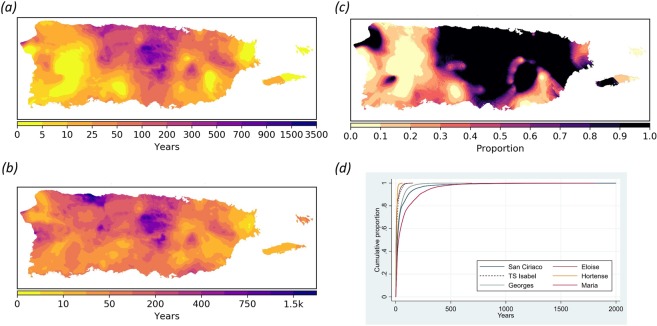
Hurricane María’s 24-h rainfall recurrence interval signature. (**a**) Spatial distribution of 24-h rainfall recurrence intervals (in years) for Hurricane María; (**b**) Overall maximum recurrence intervals of all 61 tropical cyclones from 1898 to 2017 including María; (**c**) Ratio of María’s 24-h rainfall recurrence intervals to the maximum interpolated 24-h rain recurrence interval for all 61 tropical cyclones (ratio of **a** to **b**) [value = 1 denotes where María represents the historical maximum 24-h rainfall recurrence interval]; (**d**) Cumulative distribution 24-h rainfall recurrence interval curves for the six tropical cyclones with the highest 24-h rain intensity values.

With a predicted median of 26 years (mean = 77 years), and values ranging from <1 to 1803 years, María ranks 1^st^ amongst all TCs in terms of island-wide INT-based recurrence interval (RI) (Methods; Fig. 4; Supplemental Fig. 4 and Table 4). About 23% and 8% of PR experienced 24-hr rainfall intensities that exceeded the 100-year and 250-year RI, respectively. About 1% of PR had INT values suggesting RIs greater than 600 years. The highest RIs during María occurred over the central regions of the Cordillera Central, the northern coastal valleys, with some smaller areas just southeast from Sierra de Luquillo and northwest of Sierra de Cayey.

## Conclusion

Hurricane María’s 24-hr rainfall INT and RI signatures are undoubtedly the highest in PR for a TC since at least 1898. Recent work has suggested that HM’s rainfall was indeed enhanced by global climate change^9,13^. If this holds true, HM’s rainfall signature in PR merits consideration as a new addition to the list of recent record-breaking rainfall extreme events likely attributable to global climate change^38^. HM distinguished itself from all previous TCs in PR by representing a roughly 13% increase in island-wide 24-hr rainfall rates, which is within the range of predicted increases associated to climate change both locally and worldwide. Therefore, the rain-induced impacts caused by HM in PR should serve as a reference of the potential of TC rainfall under an altered climate regime.

## Methods

### Rainfall data acquisition

Daily weather station data for 61 TCs was assembled for the period represented by weather records in PR (1898 onwards). The database includes: (a) all TC direct hits based on available track data^39^; (b) TCs for which historical accounts^40^ and reports^41,42^ describe significant rainfall and flooding; and (c) TCs from 1970 onwards that produced more than 50 mm of rainfall^43^. The number of days considered for each TC ranged from 3 to 5 and this was determined by the days when each TC’s low pressure center was within 500 km of PR’s coastline^13,15,43^. Data sources included the National Climatic Data Center (NCDC) online resources and the original Monthly Climatological Reports for PR and the West Indies available through NCDC’s Image and Publication System. The number of weather stations per TC ranged from 18 to 91 with a median of 56 stations (Supplemental Fig. 1 and Table 1). Data were compiled for total rainfall during the entire event and 24-hr average rainfall intensities in mm hr^−1^.

### Co-krigging

Weather station data were interpolated to produce continuous raster surfaces of TR and INT for the entire island. Convex-hull defined bounding polygon geometries for the weather station networks of every TC indicate that 80–94% of PR’s landmass is within these convex sets, therefore estimates for the majority of the island are based on interpolation and not extrapolation of precipitation trends (Supplemental Fig. 1 and Table 1). We employed ordinary co-krigging geostatistical methods and used elevation as a covariate variable to predict both TR and INT at unknown locations^44^. Elevation has been established as a significant factor controlling monthly and annual rainfall^45,46^ as well as TC-specific rainfall^47^. Slope and aspect were included initially but later discarded due to a lack of covariance with precipitation.

In co-kriging with one external variable, two covariance and one cross-covariance of precipitation and elevation at different spatial lags need to be calculated (Supplementary Method 1). This is accomplished by manually fitting empirical covariograms for each TC to best capture the covariance structure of the data at different spatial lags. After an initial experimentation with several covariogram models, we opted for a ‘stable’ empirical model (see SI for formal definitions) for all but four TCs. For those four, the stable model produced ‘artificial’ bands of precipitation estimates and we opted instead to employ an exponential covariogram model. The parameters used in each model, the standard error surfaces, as well as cross-validation estimates using the ‘leave-one-out’ method^48^ and corresponding root mean square error, are available upon request to the authors.

### Recurrence interval (RI)

The RI for each raster cell for every TC in our study area was calculated as follows. First, we obtained the 24-hr precipitation frequency estimates in raster format with average recurrence intervals of 1, 2, 5, 10, 25, 50, 100, 200, 500, and 1000 years from NOAA’s website^49^. At 3-second resolution (~90 m), each cell *i* in each downloaded raster stores the 24 hr rainfall value in mm h^−1^ for a particular recurrence interval. Next, an ordinary least square (OLS) model was estimated for every single cell in the raster following Eq. 1:

1 $$ln(R{I}_{j}^{i})={\beta }_{0}^{i}+{\beta }_{1}^{i}{x}_{j}^{i}+{\beta }_{2}^{i}{x}_{j}^{{i}^{2}}+{\beta }_{3}^{i}{x}_{j}^{{i}^{3}}+{u}_{j}^{i}$$

where *j* = *1*, …, *10* is the number of input values corresponding to each given recurrence interval; the superscript *i* denotes each individual raster cell (i = 1, …, 1,087,190 regressions in total), the *βs* are the coefficients to be estimated and *u* the error term. *RI* takes the value of the recurrence intervals (1, 2, 5, …, 1000) and the *x* vector contains the corresponding 24-hr rainfall intensity value. Every regression *i* therefore relies on ten INT-RI pairs of values to estimate four parameters. Once the surfaces of parameters were calculated, the estimated recurrence interval ${\widehat{RI}}^{i}$for each TC raster cell was calculated by ‘plugging-in’ each INT interpolated raster in place of the *x* vector by using GIS raster algebra (Eq. 2).

2 $${\widehat{RI}}^{i}={\hat{\alpha }}^{i}\exp ({\hat{\beta }}_{0}^{i}+{\hat{\beta }}_{1}^{i}{x}^{i}+{\hat{\beta }}_{2}^{i}{x}^{{i}^{2}}+{\hat{\beta }}_{3}^{i}{x}^{{i}^{3}})$$

The estimated β coefficients, standard errors, and R^2^’s for each cell are available upon request. Spatially explicit surfaces of the regression parameters are shown in Supplementary Material Fig. 4.

The logarithmic transformation combined with the cubic polynomial function provides an excellent approximation to the overall shape of the non-linear relationship between RI and rainfall values^50^ and guarantees that predicted RI values will always be positive. The regression R^2^ for all cells is above 0.99, an almost perfect fit (Supplemental Fig. 4). To illustrate this almost perfect fit of the regressions we present the fitted curve against the RI and rainfall values for 20 random cells within our study area (Supplemental Fig. 5).

The logarithmic transformation implies that the error term is now log-normally distributed. Therefore, OLS estimation biases the prediction of RI^51^ downward. The *α* adjustment parameter attenuates such bias^52^ and is defined as:

$${\hat{\alpha }}^{i}={n}^{-1}\mathop{\sum }\limits_{j=1}^{10}\exp ({\hat{u}}_{j}^{i})$$

where ${\hat{u}}_{j}^{i}$ are the residuals of the regression (Eq. 1). Figure 5 in the SM shows the ${\hat{\alpha }}^{i}$ parameter for every cell *i*. Because the regression fit was very good for all cells, the residuals are small and therefore the ${\hat{\alpha }}^{i}\,\,$parameters also are small. The largest correction was about 0.2%.

## Supplementary information

Supplementary Methods 1

Supplementary Methods 2

Supplemental Figures & Tables 1–5
