# Supplementary material for: Hurricane María’s Precipitation Signature in Puerto Rico: A Conceivable Presage of Rains to Come
Source: Sci Rep. 2019 Oct 30;9:15612. doi: 10.1038/s41598-019-52198-2 (PMC6821759; doi:10.1038/s41598-019-52198-2)
Supplement: Supplementary file 1 — Supplementary Methods 1 [file 41598_2019_52198_MOESM1_ESM.docx]

**Hurricane María’s Precipitation Signature in Puerto Rico: A Conceivable Presage of Rains to Come**

Carlos E. Ramos-Scharrón^[[1]](#footnote-1)^*^[[2]](#footnote-2)^, Eugenio Arima^1^

**Supplementary Methods 1- Hierarchical Cluster Analyses**

Hierarchical cluster analysis was used to characterize the (dis)similarity between each TC and provide visual clues about the strength of the clustering among different TCs. Euclidean distance between characteristics of TC *i* and *j* was used as the similarity measure

$D_{ij}=\left[ \sum_{k=1}^{5} {(x_{ik}-x_{jk})}^{2} \right]^{1/2}$

where x_k_ are descriptive statistics of TC *i* and *j* ; *k*= mean, standard deviation, median, minimum, and maximum values. Larger Euclidean distances imply more dissimilar TCs.

In addition to cluster analyses, we also implemented pairwise statistical comparisons between each TC (e.g. Tukey, Fisher’s LSD, and Duncan tests) but each storm was statistically different from one another due to the large number of observations (more than a million pixels or cells), which reduces the standard error and inflates the statistics. Therefore, cluster analysis was more informative in describing groupings of (dis)similar storms.

1. * Corresponding author: cramos@austin.utexas.edu [↑](#footnote-ref-1)
2. Department of Geography & the Environment and Lozano Long Institute of Latin American Studies, The University of Texas at Austin, Austin, TX, USA [↑](#footnote-ref-2)
