# Supplementary material for: Hurricane María’s Precipitation Signature in Puerto Rico: A Conceivable Presage of Rains to Come
Source: Sci Rep. 2019 Oct 30;9:15612. doi: 10.1038/s41598-019-52198-2 (PMC6821759; doi:10.1038/s41598-019-52198-2)
Supplement: Supplementary file 2 — Supplementary Methods 2 [file 41598_2019_52198_MOESM2_ESM.docx]

**Hurricane María’s Precipitation Signature in Puerto Rico: A Conceivable Presage**

**of Rains to Come**

Carlos E. Ramos-Scharrón^[[1]](#footnote-1)^*^[[2]](#footnote-2)^, Eugenio Arima^1^

**Ordinary Co-kriging Analyses**

The objective of kriging is to find estimates of unknown values using a weighted linear combination of known observed value. The weights (λ’s) are found by minimizing the error variance of the estimate under (unbiasedness) constraints. Let P denote maximum 24-hour precipitation intensity and A elevation. We observe P and A at locations **x**, or P(**x**_i_), A(**x**_k_), where i = 1,…n indicates the weather stations with rainfall data and k =1,…,m where we observe elevation. The goal is to predict P(**u**) or the unknown precipitation intensity at location u.

$$P\left( \mathbf{u} \right)= \sum_{i=1}^{n(\mathbf{u})} \lambda_{i}\left( \mathbf{u} \right)P\left( \mathbf{x}_{i} \right)+\sum_{k=1}^{m(\mathbf{u})} \lambda_{k}\left( \mathbf{u} \right)A\left( \mathbf{x}_{k} \right)$$

In ordinary co-kriging, we assume the means are unknown but are deemed constant within a local neighborhood. This condition can be filtered through the linear estimator above by setting two constraints on the weights^1^:

$$\sum_{i=1}^{n(\mathbf{u})} \lambda_{i}\left( \mathbf{u} \right)=1 \mathrm{and} \sum_{k=1}^{m(\mathbf{u})} \lambda_{k}=0$$

The λ’s are found through a Lagrangian constraint optimization problem. The solution to this co-kriging system includes one cross-covariance between the variables and two (co)variances:

C_PP(x,y)_ = Cov[P(x), P(y)]

C_AA(x,y)_ = Cov[A(x), A(y)]

C_PA(x,y)_ = Cov[P(x), A(y)] = C_AP(y,x)_

where x and y each denote a given set of locations. These covariance structures between known points and the locations to be predicted is unknown but can be modeled through a (semi-)variogram functional form.

Here, we modeled the cross-variances and co(variances) using the *stable* semi-variogram function γ and, in certain cases, the *exponential* model. The stable model is defined as:

$\gamma\left( \boldsymbol{h,\theta} \right)= \theta_{s}\left\lfloor1-exp\left( -3\left( \frac{|h|}{\theta_{r}} \right)^{\theta_{e}} \right) \right\rfloor$ for all *h*, where *h* is the spatial lag distance between pair of points, $\theta_{s}$ is the partial sill, $\theta_{r}$ is the range parameter and $\theta_{e}$ is an exponential parameter ranging from [0, 2]. The exponential model is:

$\gamma\left( \mathbf{h},\boldsymbol{\theta} \right)= \theta_{s}\left\lfloor1-exp\left( -\frac{3|h|}{\theta_{r}} \right) \right\rfloor$ for all *h*.

These parameters were found by non-linear weighted least square estimation that fitted the function to the average (co)variance values at each lag distance^2^.

We used ArcGIS 10.6 Geostatistical Analyst software to implement the kriging methodology described above.

**References**

^1^ Goovaerts, P. *Geostatistics for Natural Resources Evaluation*. Oxford University Press, NY, New York, 483 p. (1997).

^2^ Cressie, N.A.C. *Statistics for Spatial Data*. Wiley, NY, New York (1993).

1. * Corresponding author: cramos@austin.utexas.edu [↑](#footnote-ref-1)
2. Department of Geography & the Environment and Lozano Long Institute of Latin American Studies, The University of Texas at Austin, Austin, TX, USA [↑](#footnote-ref-2)
